# Fibulin-3 Deficiency Protects Against Myocardial Injury Following Ischaemia/ Reperfusion in *in vitro* Cardiac Spheroids

**DOI:** 10.3389/fcvm.2022.913156

**Published:** 2022-06-20

**Authors:** Poonam Sharma, Dominik Beck, Lucy A. Murtha, Gemma Figtree, Andrew Boyle, Carmine Gentile

**Affiliations:** ^1^College of Health Medicine and Wellbeing, The University of Newcastle, Callaghan, NSW, Australia; ^2^Kolling Institute of Medical Research, Royal North Shore Hospital, St Leonards, NSW, Australia; ^3^Faculty of Medicine and Health, Northern Clinical School, The University of Sydney, Sydney, NSW, Australia; ^4^Faculty of Engineering and IT, School of Biomedical Engineering, University of Technology Sydney, Sydney, NSW, Australia

**Keywords:** Fibulin-3 KO, *in vitro* cardiac models, myocardial infarction, reperfusion injury, cardiac spheroids, I/R injury

## Abstract

Myocardial infarction (MI, or heart attack) is a leading cause of death worldwide. Myocardial ischaemia reperfusion (I/R) injury typical of MI events is also associated with the development of cardiac fibrosis and heart failure in patients. Fibulin-3 is an extracellular matrix component that plays a role in regulating MI response in the heart. In this study, we generated and compared *in vitro* cardiac spheroids (CSs) from wild type (WT) and fibulin-3 knockout (Fib-3 KO) mice. These were then exposed to pathophysiological changes in oxygen (O_2_) concentrations to mimic an MI event. We finally measured changes in contractile function, cell death, and mRNA expression levels of cardiovascular disease genes between WT and Fib-3 KO CSs. Our results demonstrated that there are significant differences in growth kinetics and endothelial network formation between WT and Fib-3 KO CSs, however, they respond similarly to changes in O_2_ concentrations. Fib-3 deficiency resulted in an increase in viability of cells and improvement in contraction frequency and fractional shortening compared to WT I/R CSs. Gene expression analyses demonstrated that Fib-3 deficiency inhibits I/R injury and cardiac fibrosis and promotes angiogenesis in CSs. Altogether, our findings suggest that Fib-3 deficiency makes CSs resistant to I/R injury and associated cardiac fibrosis and helps to improve the vascular network in CSs.

## Introduction

Myocardial infarction (MI) or heart attack is the leading cause of death across the world (1). Prolonged severe myocardial ischaemia initiated by the ruptured epicardial coronary artery plaque that causes the occlusion of the coronary artery leads to MI (2). Prompt reperfusion of the ischaemic myocardium using therapeutic and/or surgical interventions is necessary to prevent and protect the heart from the detrimental effects of MI. However, reperfusion itself induces pathophysiological changes leading to irreversible injury typically known as myocardial ischaemia reperfusion (I/R) injury (2, 3). Reperfusion also causes irreversible damage in endothelial cells (ECs) which are usually resistant to acute MI and leads to EC proliferation (4). This ultimately leads to the activation of proangiogenic and vascular destabilizing factors in the infarct zone (5, 6). Myocardial I/R injury also triggers fibroblast infiltration leading to cardiac fibrosis in the ischaemic area (2). Current *in vivo* and *in vitro* models of MI failed to address myocardial I/R injury (1). Therefore, it has become difficult to translate the laboratory findings into the clinic. Another potential drawback that causes a lack of treatments for MI patients is the unavailability of novel molecular and cellular targets. Targeting new epigenetic molecules or genes could present better therapeutic results than the pre-existing interventions. Therefore, there is a need to find novel targets for the treatment of MI patients.

Fibulin-3 (Fib-3) is an extracellular matrix (ECM) glycoprotein encoded by the gene EFEMP1 in both mice and humans (7). Fib-3 lacks the RGD binding motif for integrins (8). It is abundantly present across the body including the lungs, ovary, kidney, skeletal muscle, and the adult human heart (9, 10). Fib-3 is predominantly produced by ECs and fibroblasts in the heart (11). Fib-3 is present in the vessel walls, especially in small capillaries and the aorta; however, the large myocardial vessels and heart valves are devoid of Fib-3 (9, 12, 13). The genetic variants of Fib-3 present modified vein wall elasticity or reduced strength that leads to the occurrence of chronic venous disease and varicose veins and in some cases a splice site alteration leading to loss of TIMP-3 interacting site of Fib-3 (14, 15). This highlights Fib-3's role in ECM enzyme maintenance and may also show the role of Fib-3 in angiogenesis and vascular remodeling resulting from changes in endothelial cell behavior (16). Fib-3 plays a role in essential hypertension, where it was reported that decreased serum levels of Fib-3 are correlated with severe endothelial dysfunction (17). Fib-3 is upregulated in non-ischaemic and ischaemic heart failure patients (18). Fib-3 is also linked with diastolic blood pressure and pulse pressure suggesting it may play a role in cardiovascular system regulation (19). Altogether, these studies support the role played by Fib-3 on endothelial function.

Deficiency of Fib-3 in mice has been shown to result in reduced reproductivity, early aging with a shorter lifespan, decreased body mass, reduced hair growth, spine deformity, and decreased bone density suggesting a prominent role in regulating the integrity of connective tissue and aging (20, 21). The first identification of upregulated Fib-3 was shown in senescent human fibroblasts of a patient with Werner syndrome which causes premature aging (20–22). Later, the upregulation of Fib-3 was found to be linked with the hypertensive vascular remodeling in the thoracic aortas of spontaneously hypertensive rats (23). A more recent study has shown that Fib-3 is upregulated in fibrotic and infarcted mouse hearts suggesting its potential role as a target for novel therapeutics for infarcted patients (24).

We hypothesized that Fib-3 may be a potential target to study myocardial I/R injury and could be potentially used to develop better therapeutic interventions for MI patients. In this study, we evaluated the role(s) played by Fib-3 in our three dimensional (3D) *in vitro* I/R injury model using cardiac spheroids (CSs) generated from neonatal mouse cardiac cells, based on our recently published studies (1). Therefore, CSs generated from cardiac cells isolated from either wild type (WT) and Fib-3 knockout (KO) neonatal mice were used to study I/R injury in *in vitro* conditions.

## Materials and Methods

### Generation of Neonatal Mouse CSs

**Figure 1:**
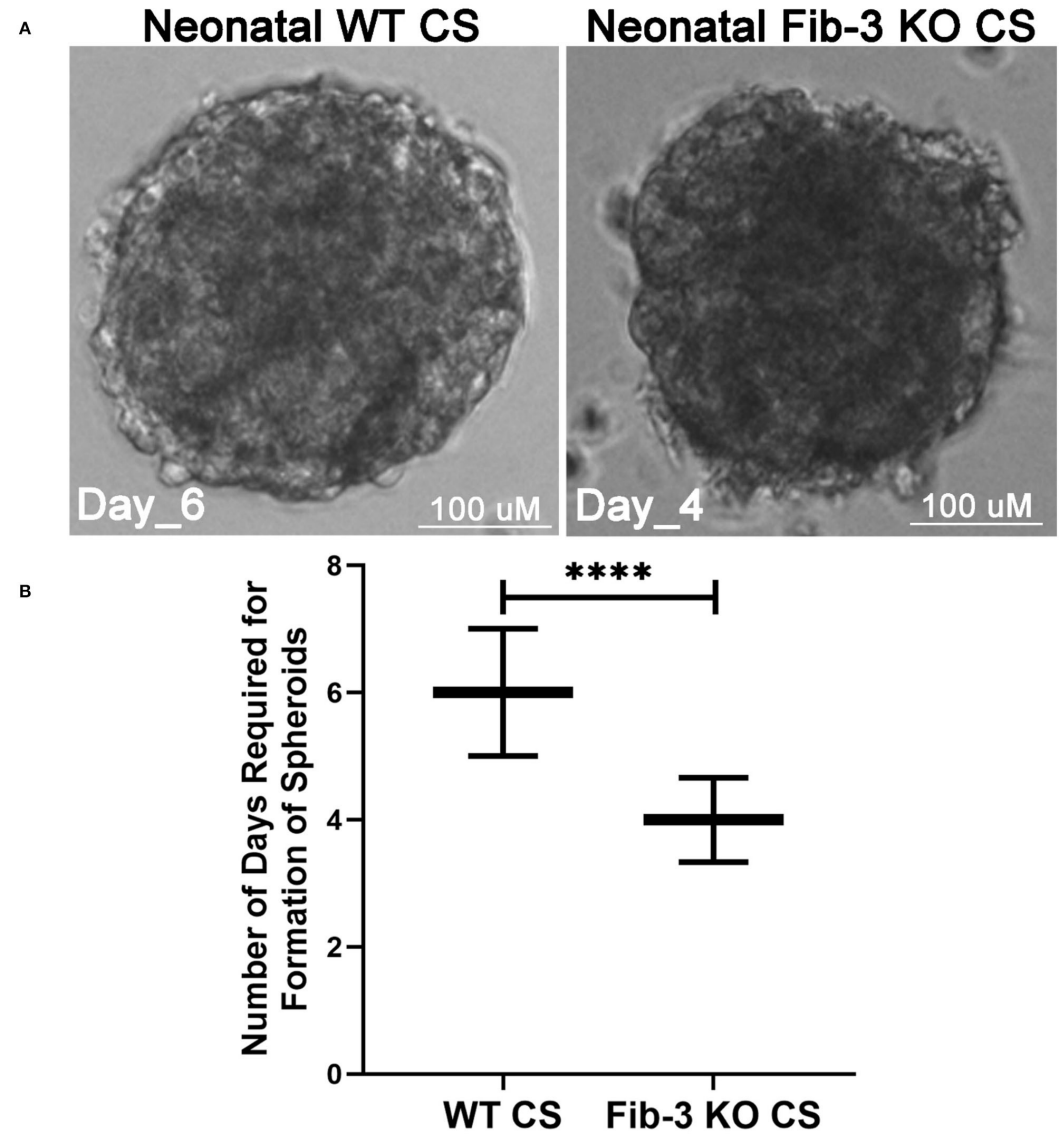
Fib-3 KO CSs form faster than WT CSs. **(A)** Representative images of WT and Fib-3 KO CSs at day 4. **(B)** Statistical analyses of CS formation in WT vs. Fib-3 KO CSs. Unpaired *t-*test, *p* < 0.0001 = ^****^. Error bars represent the mean ± SD (*n* = 384).

Neonatal mouse hearts were isolated according to our laboratory's established protocol under the protocol number RESP_17/55 from the Animal Ethics Committee at the Northern Sydney Local Health District St Leonards, NSW, Australia (1). Briefly, murine whole hearts were isolated from 1 to 5-day old WT and Fib-3 KO mice, then cut into smaller pieces and digested enzymatically using GentleMACS dissociator. The enzyme reaction was blocked by adding DMEM media containing 10% FBS, single cells were then isolated using a cell strainer on top of the 50-mL tube and then centrifuged at 300 g, for 5 min at 4°C. The cell pellet was resuspended into DMEM media containing 10% FBS. CSs were generated by co-culturing 30,000 mice cardiac cells (immediately after their isolation) in 15-μL hanging drop cultures containing DMEM media containing 10% FBS, using Perfecta 3D ® 384-well-hanging drop plates (3D Biomatrix, Ann Arbor, MI, USA). Media was replaced every other day until all the cardiac cells aggregate together as one sphere (a completely formed CS) (Figure 1).

### Confocal Imaging and 3D Rendering Analysis of CSs

Both WT and Fib-3 KO CSs were fixed with 4% paraformaldehyde for an hour at room temperature to stop the cell distribution process, permeabilized in PBS/0.01% sodium azide (PBSA) containing 0.02% Triton-X-100 for 60 min, blocked with a 3% bovine serum albumin/PBSA solution, and then incubated with appropriate primary (15 μg/mL) and secondary (10 μg/mL) antibodies at 4 °C (18 h). For cell behavior analysis, fixed CSs were exposed to primary antibodies against cardiac troponin T and CD31, to stain CMs and ECs, respectively (1). Nuclei were stained with Hoechst staining (Invitrogen, Carlsbad, CA). CSs were mounted on a glass slide using Vectashield Mounting Medium (Vector Laboratories, Burlingame, CA). Fluorescent imaging used a Zeiss LSM 800 Laser Confocal Microscope (Carl Zeiss AG, Oberkochen, Germany). Optical sectioning along the Z axis was performed, and the images collapsed into a single focal plane using the manufacturer's software. Images were processed using Adobe Photoshop CC (Adobe Systems, Inc., San Jose, CA). 3D rendering analysis was performed on the confocal images using Imaris software which allows conversion of each pixel of a z-stack into a 3D voxel (3D sectioning) to obtain quantitative information from the image (1, 25).

### Establishment of Myocardial I/R Injury in CSs

Following our previously published protocol, CSs were collected in 96 well-plate from hanging drop culture plate and incubated with 10 μM image-IT (a reversible hypoxic dye for intracellular hypoxia) for 1 h at 37^0^C, then exposed to normoxia (5% O_2_, 24 h), followed by hypoxia (0% O_2_, 20 h), followed by normoxia (5% O_2_, 17 h) in an EVOS FL Auto Live imaging system (Life Technologies) to induce I/R injury in CSs (1). Control CSs were kept in the incubator in normoxic conditions for the whole duration of the procedure (24 + 20 + 17 h) and stained with image-IT to confirm no changes in intracellular O_2_ concentrations.

### Cell Viability and Death Analysis

Cell viability and death were evaluated by incubating CSs with calcein-AM and ethidium homodimer (staining live and dead cells, respectively) as previously described (Invitrogen LIVE/DEAD Viability/Cytotoxicity Kit) (25, 26). Nuclei were stained with Hoechst stain. Stained CSs were imaged using a Evos FL Auto microscope. Images were processed using Adobe Photoshop CC (Adobe Systems, Inc., San Jose, CA) and NIH ImageJ software to obtain black and white ratios of the CSs, which were then used for fluorescence quantification.

### Contractile Activity of CSs

The contraction frequency and fractional shortening of the CSs was evaluated using an IonOptix system including a video-based edge-detection. Briefly, CSs were placed on the water bath of the microscope and the videos of CSs were recorded using the connected Ionoptix software. Thereafter the contraction frequency (number of contractions per minute) and fractional shortening percentage (total length - length of contraction)/total length x 100) of the CSs was calculated manually.

### MRNA Extraction and QPCR Analyses

RNA was isolated from control and I/R CSs generated from WT and Fib-3 KO mice using RNeasy Plus Mini Kit (Qiagen) following manufacturer's guidelines. RNA quality was measured using Nanodrop machine. Reverse transcription was performed on the RNA obtained using RT2 First Strand Kit, and the resulting cDNA (20 μL) was diluted with 91 μL of water and used as polymerase chain reaction (PCR) template. Diluted cDNA was then mixed with 2 × RT2 SYBR Green Mastermix (Qiagen) and water and transferred on the mouse and human cardiovascular disease PCR array (PAMM-174Z and PAHS-174Z, Qiagen). PCR was performed using Quantstudio 12K Flex PCR machine with a 384 block. Ct values were exported and analyzed using Qiagen web-based analysis tools. Three biological repeats were performed. The normalized differentially expressed fold change values (log2 fold change ≥ |1|, adjusted *P* < 0.05) of all the genes of the array obtained from Qiagen web-based analysis were used to produce an unsupervised hierarchical clustering heat map in Partek Genomics Suite software (version 7.0) (Partek Inc., St. Louis, MO, USA).

### Statistical Analyses

Data presented in this manuscript were analyzed using Graphpad Prism software, mean ± standard deviation (SD) and appropriate unpaired *t-*test according to distribution and sample variance. One-way ANOVA with Tukey *post-hoc* test, Two-way ANOVA with Sidak's multiple comparison test was used for comparisons of multiple groups. Significance was set to *p* < 0.05. For single gene expression analysis, fold changes [calculated as 2^∧^(-Avg.ΔΔCt)] were analyzed based on the online software from Qiagen. A minimum of *n* = 3 biological replicates were used per group.

## Results

### WT and Fib-3 KO Cardiac Spheroids Present Differences in Tissue Kinetics and Endothelial Cell Network Formation

**Figure 2:**
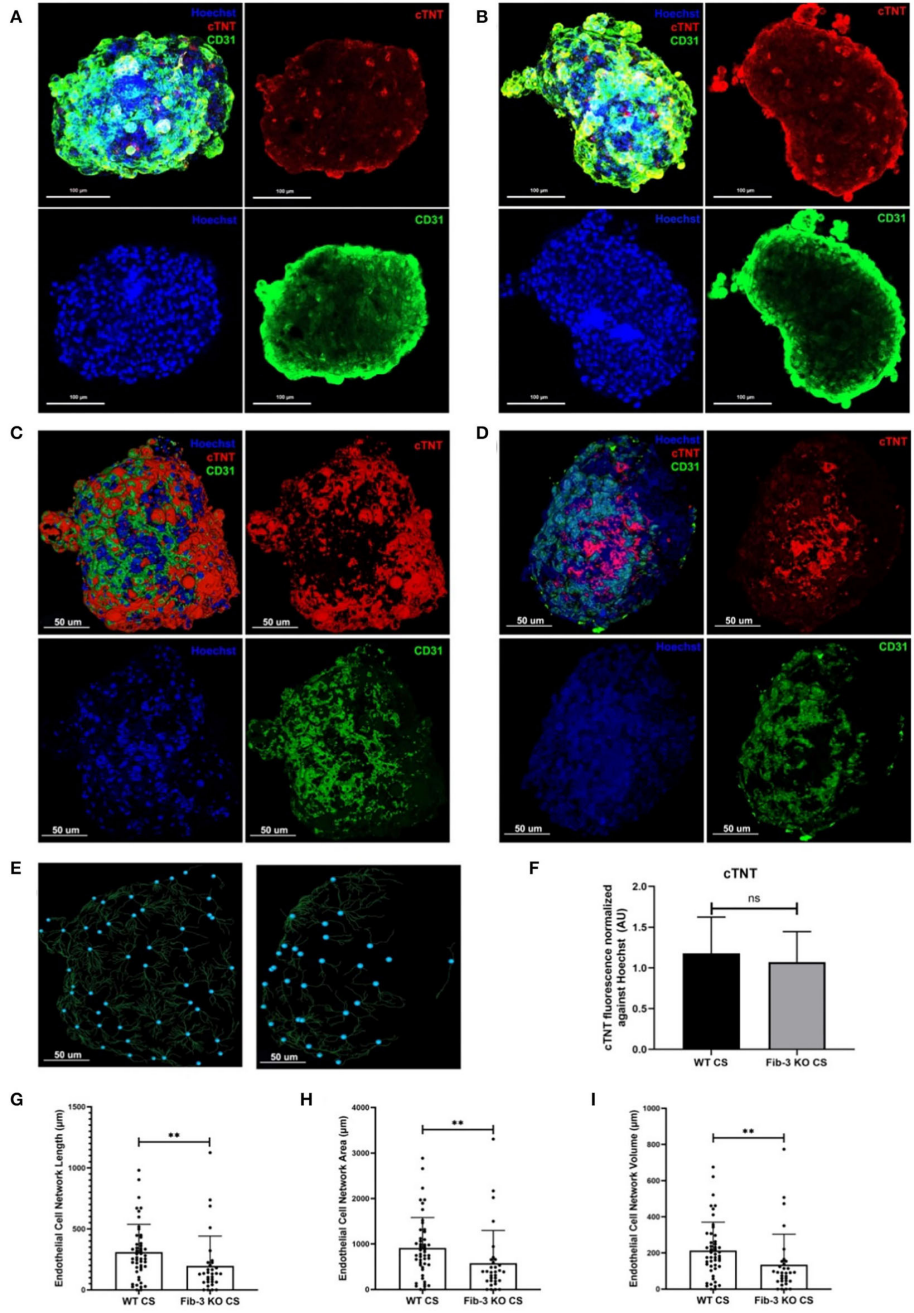
Fib-3 KO CS demonstrate reduced CD31-positive endothelial cells compared to WT CSs. **(A, B)** Collapsed Z-stacks of confocal images of WT and Fib-3 KO CS stained with antibodies against CD31 for endothelial cells (green), cTNT for cardiomyocytes (red) and Hoechst for nuclei (blue). **(C, D)** 3D rendering analyses of confocal images of WT **(A)** and Fib-3 KO CS **(B)**, respectively. **(E)** 3D representation of endothelial cell network formation in WT and Fib-3 KO CS (blue dots represent foci of the endothelial cell network formed within each CS). **(F)** Statistical analyses of expression of cTNT normalized against Hoechst stain in WT and Fib-3 KO CSs, (*n* = 5). **(G–I)** Statistical analyses of endothelial cell network length, area and volume in WT and Fib-3 KO CSs. Unpaired *t*-test, *p*>0.05= ns, *p* < 0.01 = **. Error bars represent the mean ± SD (*n*>32).

In order to evaluate any differences in *in vitro* CS from WT and Fib-3 KO cardiac cells, we first evaluated any changes in spheroid kinetics until complete spheroid formation. Our analysis showed that Fib-3 deficient cells were significantly faster in forming CSs compared to WT cells (4 vs. 6 days, Figures 1A,B and Supplementary Figure 8). Our confocal analyses demonstrated no difference in cTNT-positive cardiac myocytes (Figure 2F). Our 3D rendering analyses confirmed a significant decrease in the vascular network formation measured as length, area and volume of CD31^+^ endothelial cells in Fib-3 KO CSs compared to WT CSs (Figures 2E,G–I, and Supplementary Videos 1, 2). Altogether, our findings suggest that absence of Fib-3 has a direct effect on the endothelial cell population, while it does not affect cardiac myocytes (Figure 2).

### I/R Injury Model in Fib-3 KO and WT CSs

**Figure 3:**
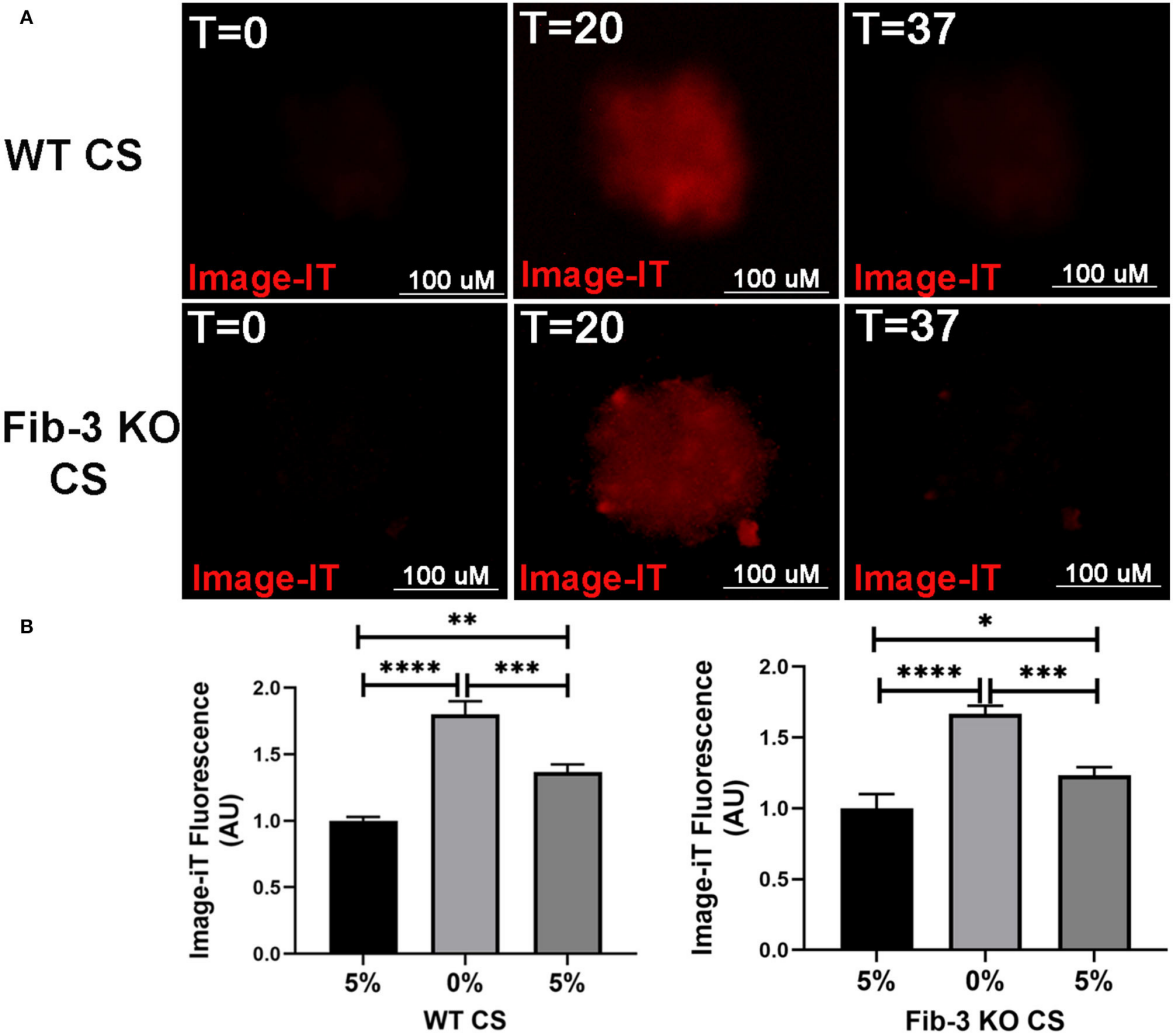
Establishment of hypoxic/normoxic damage in Fib-3 KO and WT CSs. **(A)** Epifluorescent images of a representative CS treated with Image-iT (become fluorescent red in lower oxygen conditions) that was pre-incubated at 5% O_2_ for 24 h (T = 0), and then exposed to 0% O_2_ for 20 h (T = 20) and 5% O_2_ for additional 17 h (T = 37). **(B)** Statistical analysis of changes i n Image-iT fluorescence between 5% (T = 0), 0% (T = 20) and 5% (T = 37) between WT and Fib-3 KO CSs. One-way ANOVA with Tukey *post-hoc* test was used *f* or comparison of all groups, *p* < 0.05 = *, *p* < 0.01 = **, *p* < 0.001 = ***, and *p*>0.0001 = ****. Error bars represent the mean ± SD (*n* = 3).

To evaluate the effect of pathophysiological changes in O_2_ concentration typical of an MI event, CSs generated from both WT and Fib-3 KO neonatal mice were incubated with 10 μM image-IT in presence of normoxic (5% O_2_) and hypoxic (0% O_2_) conditions to induce I/R injury in CSs, according to our recently published protocol (1). Then, CSs were analyzed over time for changes in fluorescence intensity of image-IT (where red fluorescence was used to identify that intracellular hypoxia was achieved) following changes in intracellular O_2_ concentrations. Our statistical analyses of image-IT fluorescence measurements at different times (T0, T20 and T37), confirmed that both Fib-3 KO and CS sensed changes in levels of O_2_ concentration at similar times (Figure 3).

### Fib-3 KO CSs Prevent Cell Toxicity Following I/R Injury

**Figure 4:**
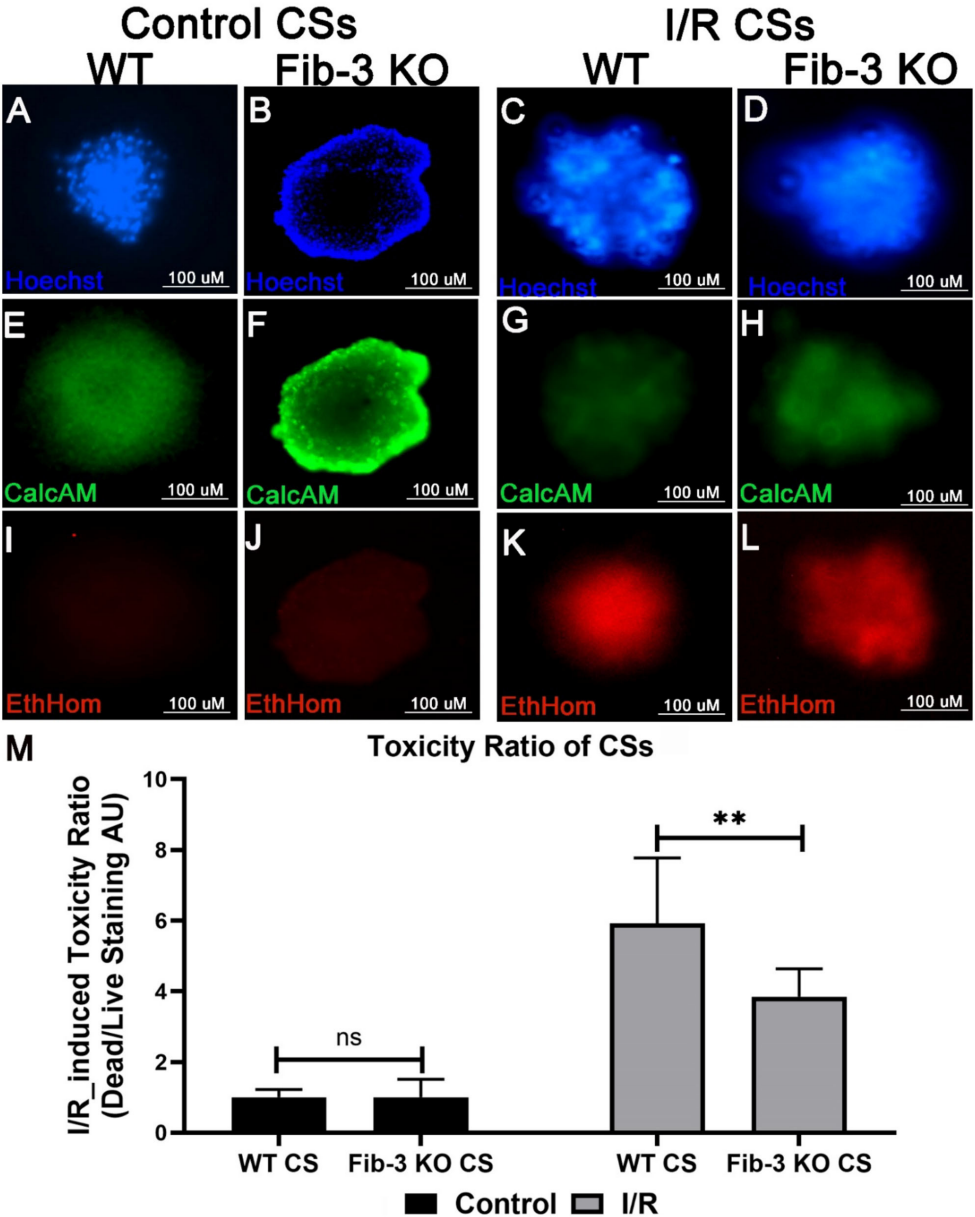
Fib-3 KO CSs are less sensitive to changes in oxygen concentrations compared with WT CSs. **(A–L)** Representative images of control and I/R WT and Fib-3 KO CSs stained with calcein-AM (green), ethidium homodimer (red) and Hoechst stain (blue), staining live, dead, and total cells, respectively. **(A–D)** stained with Hoechst, **(E–H)** with calcein-AM, and **(I–L)** and ethidium homodimer. **(M)** Statistical analyses of toxicity ratios for control and I/R CSs. Two-way ANOVA with Sidak's multiple comparison test, *p*>0.05= ns and *p* < 0.01 = **. Error bars represent the mean ± SD (*n*>6).

In order to evaluate changes in cell viability and death in both WT and Fib-3 KO CSs, toxicity ratios were calculated by measuring the fluorescence of calcein-AM and ethidium homodimer, staining dead and live cells, respectively (Figures 4A–L). Our statistical analyses showed a statistically significant increase in cell death compared to control (normoxic) CSs for both Fib-3 KO and WT CSs. Fib-3 KO CSs demonstrated significantly reduced cell death following I/R injury compared to WT CSs (Figure 4M).

### Fib-3 KO and WT CSs Present Reduced Contraction Frequency and Fractional Shortening Following I/R Injury

**Figure 5:**
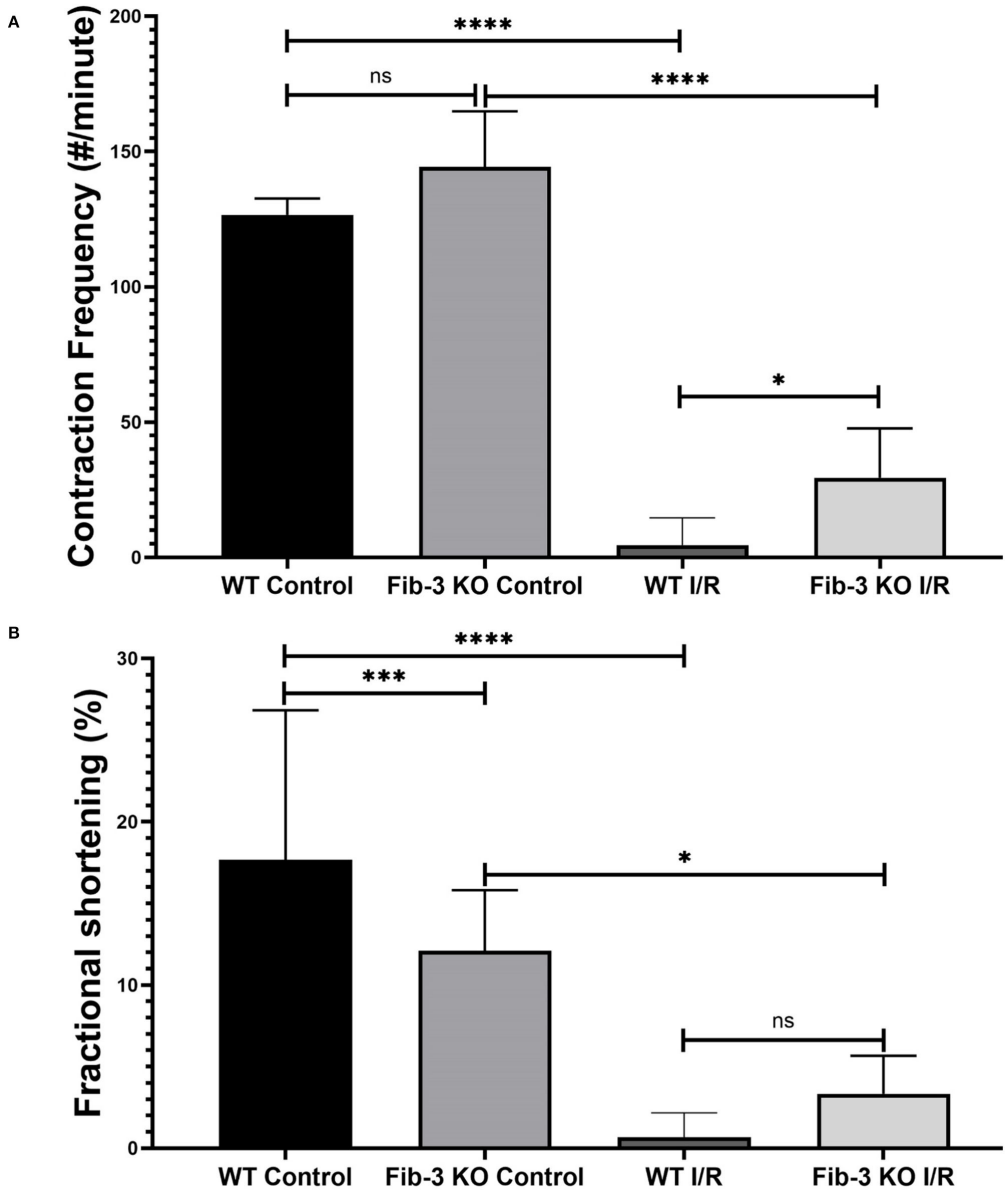
I/R conditions decrease contraction frequency and fractional shortening in CSs. Statistical analysis of contractile frequency **(A)** and fractional shortening **(B)** of control and I/R CSs from both WT and Fib-3 KO mouse optimized under Olympus microscope of Ionoptix system. One-way ANOVA with Tukey *post-hoc* test was used for comparison of all groups, *p* > 0.05 = ns, *p* < 0.05 = ^*^, *p* < 0.001 = ^***^ and *p* < 0.0001 = ^****^. Error bars represent the mean ± SD (*n* > 5).

To evaluate changes in contractile activity following I/R conditions, we measured contraction frequency and fractional shortening for both WT and Fib-3 KO CSs. We measured that both control WT and Fib-3 KO CS presented 127 and 144 contractions per minute, respectively. While the majority of WT I/R CS stopped contracting, Fib-3 KO CSs were still able to contract following injury with 30 ± 18 contractions per minute (Figure 5A). Fractional shortening was significantly decreased in control Fib-3 KO compared to WT, which was maintained following I/R injury (Figure 5B). Altogether, our findings suggest that Fib-3 deficiency protected against I/R injury-induced changes in contractile function (Figures 5A,B; Supplementary Videos 3–6).

### Changes in Relative Expression of Cardiovascular Genes in WT *vs*. Fib-3 KO CSs Following I/R Injury

**Figure 6:**
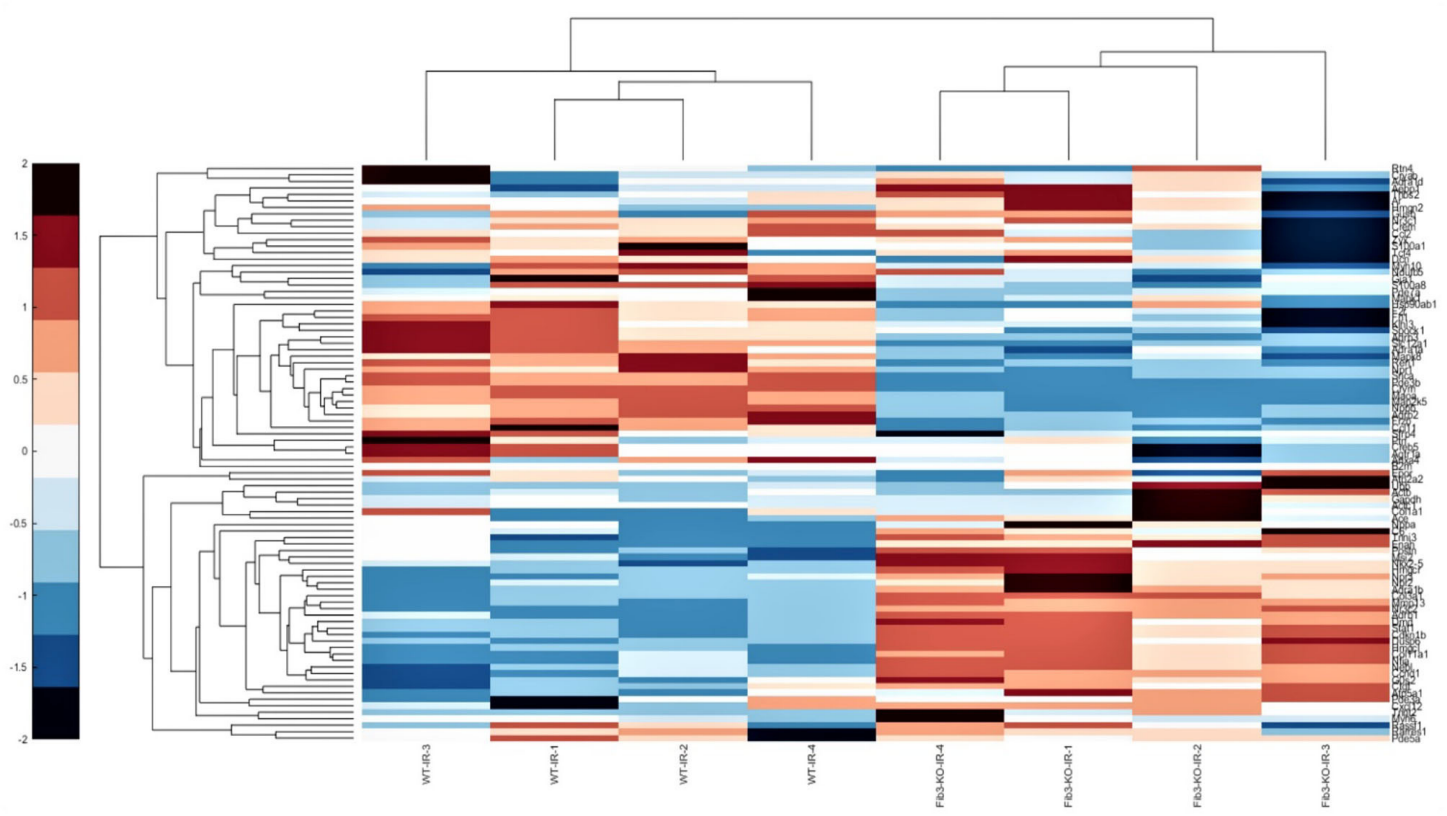
Fib-3 KO and WT CSs differentially respond to changes in oxygen concentrations. Heat map of the differential expression of cluster of cardiovascular genes of WT and Fib-3 KO I/R CSs moving from right to left. The color bar on the right side indicates the intensity of expression.

**Figure 7:**
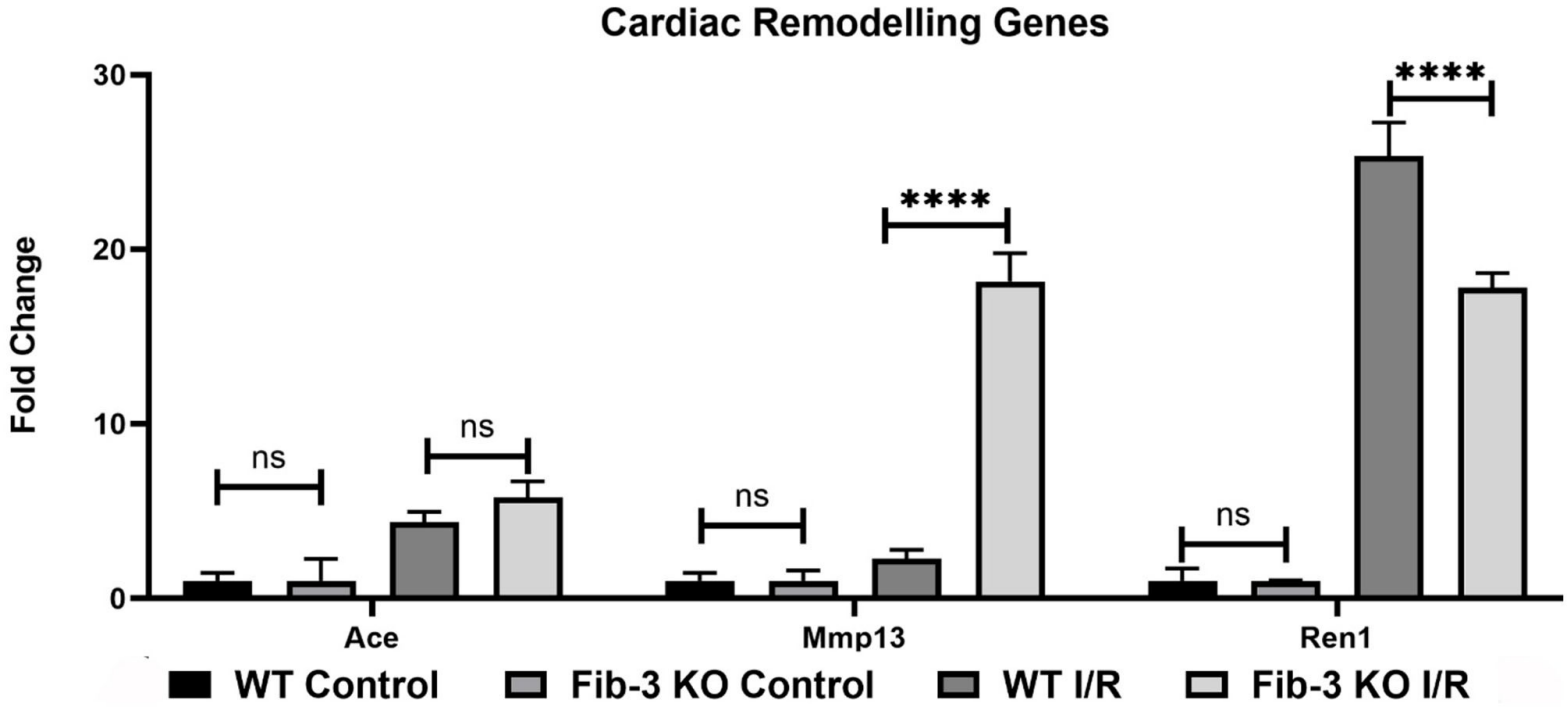
Changes in cardiac remodeling genes in WT and Fib-3 KO CSs following I/R injury. Relative expression of Ace, Mmp13 and Ren1 in WT and Fib-3 KO CSs. Two-way ANOVA with Sidak's multiple comparison test, *p* > 0.05 = ns and *p* < 0.0001= ****. Error bars represent the mean ± SD (*n* = 4).

**Figure 8:**
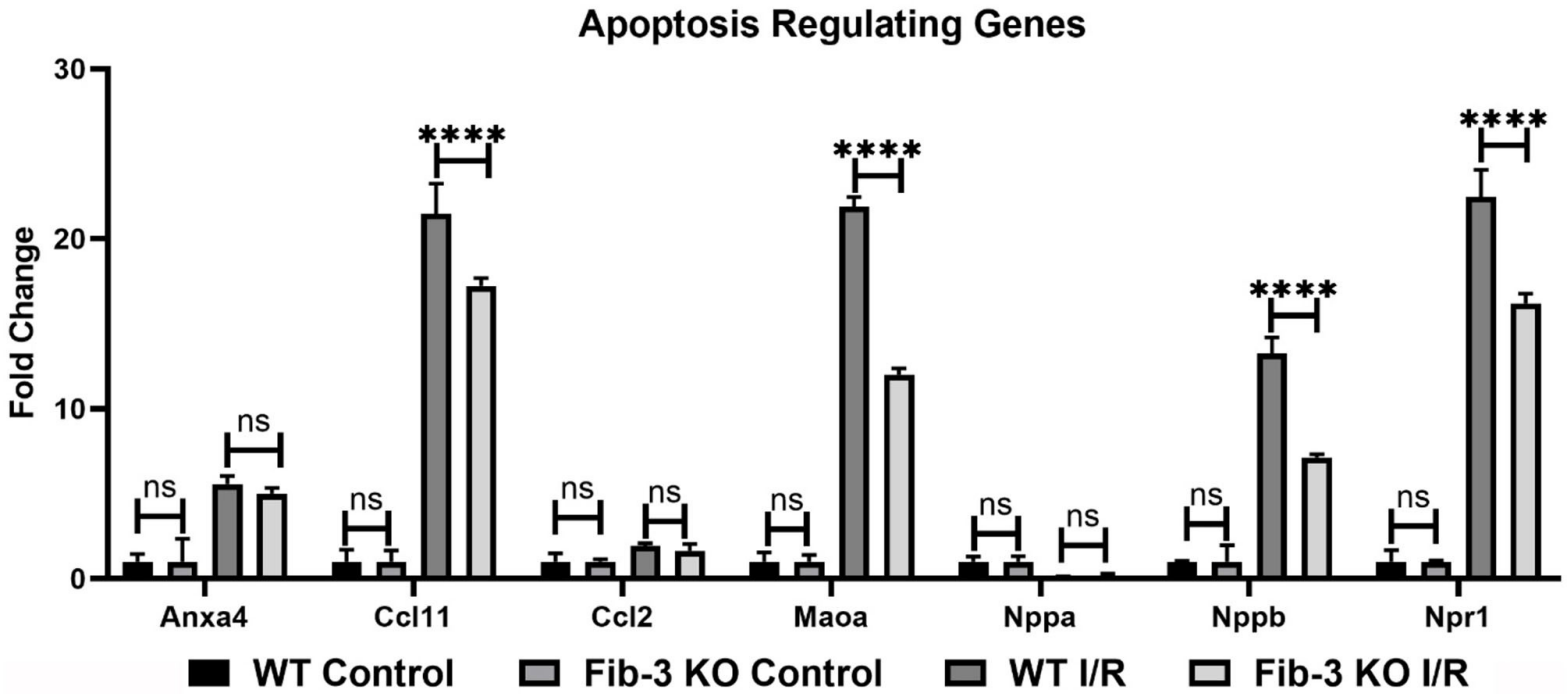
Changes in apoptosis regulating genes in WT and Fib-3 KO CSs following I/R injury. Relative expression of Anxa4, Ccl11, Ccl2, Maoa, Nppa, Nppb and Npr1 in WT and Fib-3 KO CSs. Two-way ANOVA with Sidak's multiple comparison test, *p* > 0.05 = ns and *p* < 0.0001= ^****^. Error bars represent the mean ± SD (*n* = 4).

**Figure 9:**
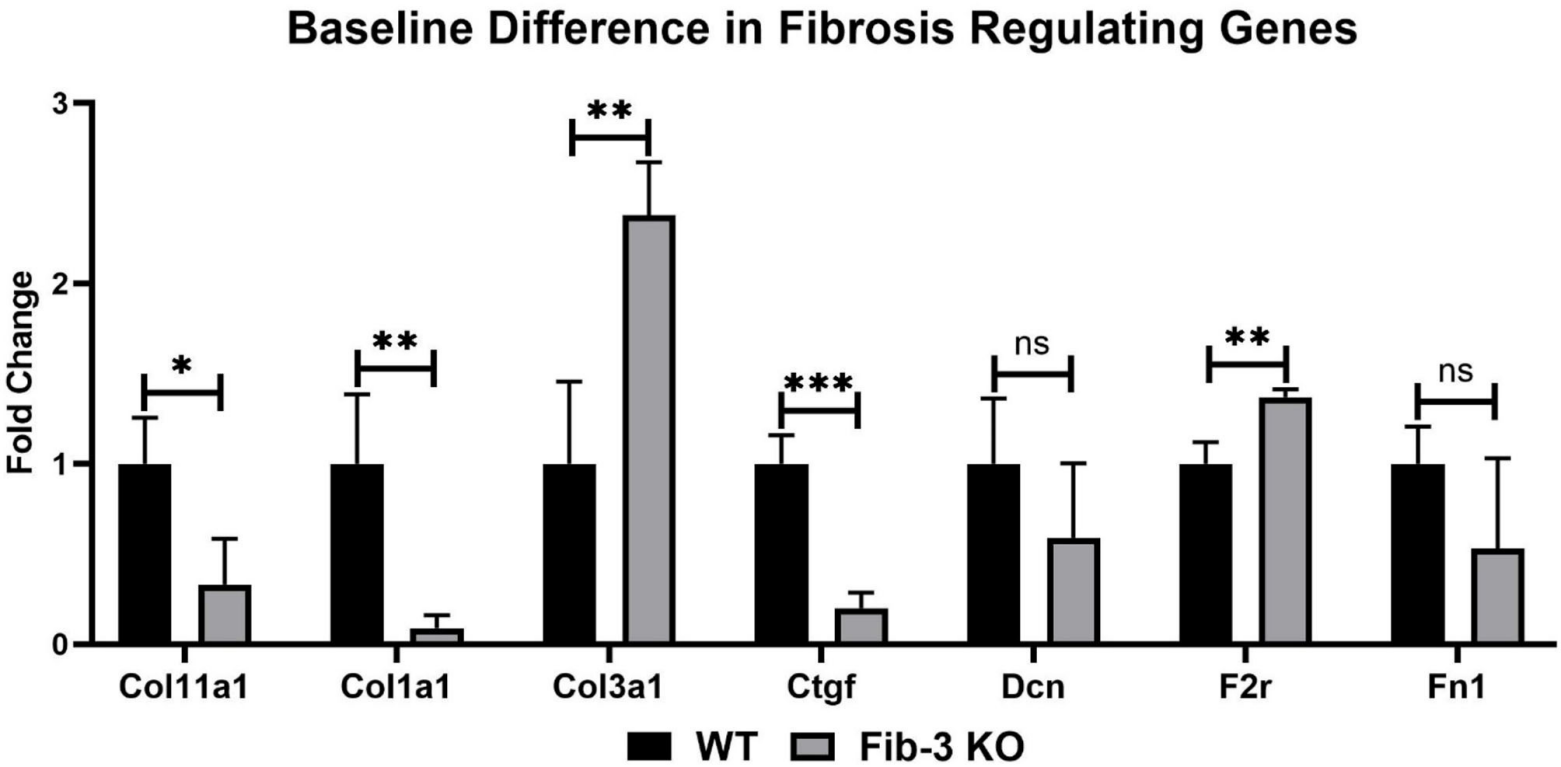
Changes in fibrotic genes in non-infarcted WT and Fib-3 KO CSs. Relative expression of Col11a1, Col1a1, Col3a1, Ctgf, Dcn, F2r and Fn1 in WT and Fib-3 KO CSs. Unpaired *t-*test, *p* > 0.05 = ns, *p* < 0.05 = *, *p* < 0.01 = ** and *p* < 0.001 = ***. Error bars represent the mean ± SD (*n* = 4).

**Figure 10:**
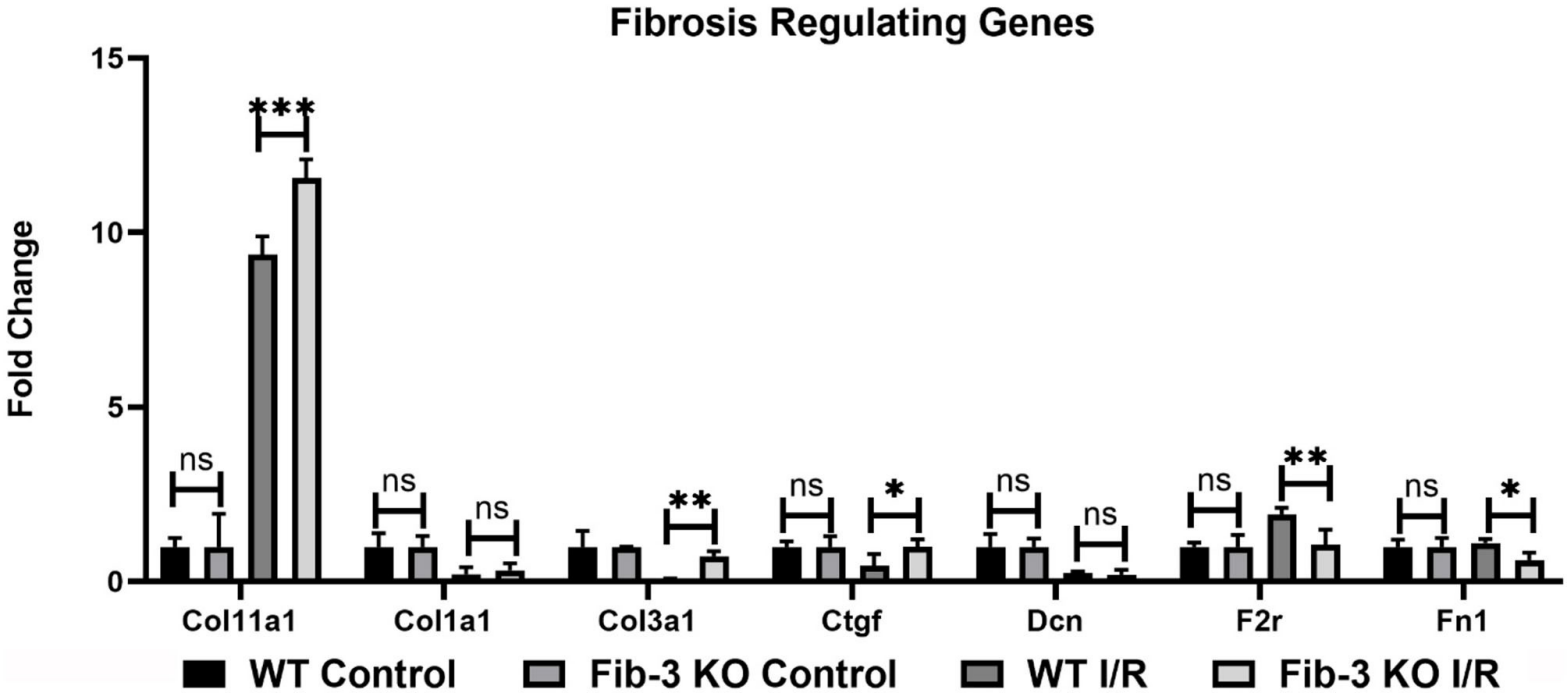
Changes in fibrotic genes in WT and Fib-3 KO CSs following I/R injury. Relative expression of Col11a1, Col1a1, Col3a1, Ctgf, Dcn, F2r and Fn1 in WT and Fib-3 KO CSs. Two-way ANOVA with Sidak's multiple comparison test, *p* > 0.05 = ns, *p* < 0.05 = *, *p* < 0.01 = ** and *p* < 0.001 = ***. Error bars represent the mean ± SD (*n* = 4).

To further evaluate how Fib-3 deficiency plays a role in cardiovascular damage from the molecular point of view, we evaluated expression level changes of cardiovascular disease-related genes following an I/R event for both WT and Fib-3 KO CSs (Table 1; Figures 7–10; Supplementary Figures 1–7). Fold change values of all the genes from both WT and Fib-3 KO I/R CSs compared to respective control CSs were then used to generate heat map to show hierarchal differential gene expression (Figure 6). We found that, among all the genes analyzed, cardiac remodeling genes (*i.e*., angiotensin-I-converting enzyme (Ace), matrix metallopeptidase 13 (Mmp13) and renin 1 structural (Ren1), were consistently upregulated in both WT and Fib-3 KO I/R CSs compared to controls (Table 1). When comparing I/R CSs, Mmp13 was significantly increased, while Ren1 was significantly decreased in Fib-3 KO compared to WT CSs (Figure 7).

**Table 1:** Expression of cardiovascular genes following I/R injury.

**QPCR analyses of cardiovascular genes for I/R injury evaluation**					
		**WT CSs**		**Fib-3 KO CSs**	
**Classification of genes**	**Genes**	**Fold change**	***P*** **-value**	**Fold change**	***P*** **-value**
Sarcomeric genes	Actc1	0.04586075	***	0.118825	***
	Myh10	2.86757775	****	2.355	**
	Myh6	0.052792	**	0.08575	**
	Tnni3	0.140802	****	0.3475	****
	Tnnt2	0.082061	**	0.2975	**
Calcium transporting genes	Atp2a2	0.0580725	****	0.09175	****
	Atp5a1	0.21521475	****	0.63	*
Cell cycle genes	Ccnd1	3.0402745	ns	6.415	****
	Rarres1	14.85651867	****	16.39	****
Cardiac remodeling genes	Ace	4.368497	***	5.775	****
	Mmp13	2.2740775	ns	18.1625	****
	Ren1	25.35145925	****	17.7975	****
Apoptotic genes	Anxa4	5.5666525	****	4.995	****
	Ccl11	21.4988795	****	17.228	****
	Ccl2	1.9312525	**	1.655	ns
	Maoa	21.888545	****	12.01	****
	Nppa	0.08316425	***	0.195	**
	Nppb	13.248215	****	7.1375	****
	Npr1	22.4866	****	16.19333333	****
Fibrotic genes	Col11a1	9.37192075	****	11.56	****
	Col1a1	0.215887	**	0.33	*
	Col3a1	0.0536505	***	0.725	ns
	Ctgf	0.448494	ns	1.005	ns
	Dcn	0.25672775	**	0.1925	**
	F2r	1.92838675	**	1.0625	ns
	Fn1	1.10904125	ns	0.61	ns
Adrenergic receptor genes	Adra1a	24.00915	****	17.495	****
	Adra1b	2.44506525	ns	4.94	***
	Adra1d	21.65162775	****	21.14725	****
	Adrb1	2.42792	****	17.15225	****
	Adrb2	29.1754925	****	18.61375	****
Map kinases	Map2k5	21.335	****	5.5075	**
	Mapk1	1.897011	ns	1.27625	ns
	Mapk8	5.60641075	****	3.52	***
Phophodiestrases	Pde3a	2.282268	ns	2.73	ns
	Pde3b	74.720255	****	17.3475	****
	Pde5a	7.623880833	**	8.72	***
	Pde7a	9.8827485	****	8.6225	****

Relative expression of sarcomere genes, calcium transporting genes, cell cycles genes, apoptotic genes, fibrotic genes and signal transduction genes in WT and Fib-3 KO CSs. The blue color represents downregulated genes and red represents the upregulated genes. Unpaired t-test, p > 0.05 = ns, p < 0.05 = ^*^, p < 0.01 = ^**^, p < 0.001 = ^***^ and p < 0.0001 = ^****^, (n = 4).

Our mRNA expression analyses of apoptosis regulating genes showed that these [*i.e*., annexin A4 (Anxa4), chemokine (C-C motif) ligand 11 (Ccl11), and 2 (Ccl2), monoamine oxidase A (Maoa), natriuretic peptide type B (Nppb) and natriuretic peptide receptor 1 (Npr1)] were all significantly upregulated following myocardial injury in WT and Fib-3 KO CSs compared to controls (Table 1). However, natriuretic peptide type A (Nppa) was significantly downregulated in both conditions (Table 1). When comparing I/R CSs, apoptotic genes such as Ccl11, Maoa, Nppb and Npr1 were significantly decreased in Fib-3 KO CSs compared to WT CSs (Figure 8). When evaluating fibrosis regulating genes [such as collagen, type XI, alpha 1 (Col11a1); type I, alpha 1 (Col1a1); type III, alpha 1 (Col3a1), connective tissue growth factor (Ctgf), decorin (Dcn), coagulation factor II (thrombin) receptor (F2r) and fibronectin 1 (Fn1)] in non-infarcted WT and Fib-3 KO CSs, we found a significant downregulation in Col11a1, Col1a1, Ctgf expression and a significant upregulation in Col3a1 and F2r expression in Fib-3 KO CSs compared to WT CSs (Figure 9). The changes in the expression of these genes were consistent between I/R WT and Fib-3 KO CSs (Table 1). When comparing I/R CSs, our statistical analyses showed a significant increase in expression of Col11a1, Col3a1, Ctgf in Fib-3 KO CSs compared to WT CSs. On the contrary, F2r and Fn1 were significantly downregulated in Fib-3 KO CSs compared to WT CSs (Figure 10).

## Discussion

In this study, we have used our previously established myocardial I/R injury protocol to study the effects of knocking down an extracellular matrix glycoprotein, Fib-3. We generated CSs from WT and Fib-3 KO neonatal cardiac cells and evaluated their response in terms of cell viability, vascular network formation and gene expression levels in control and I/R conditions. Our study showed that both WT and Fib-3 KO CSs were similar in terms of responding to pathophysiological changes in O_2_ concentration, toxicity ratio and changes in mRNA expression levels of cardiovascular disease related genes following I/R injury. We also showed differences in growth kinetics, endothelial cell network formation, and contractile function of WT and Fib-3 KO CSs. We demonstrated that Fib-3 KO CSs form faster than WT CSs. While the reason behind faster formation of Fib-3 KO CSs has not been the focus of this study, it could potentially compared with a faster aging process typical of Fib-3 KO mice (21). Our 3D rendering analyses of confocal images showed that knocking down Fib-3 alters vascular endothelial network formation as evaluated by CD31-stained endothelial cell network length, area and volume within CSs. This is consistent with a previous study which showed that Fib-3 KO mice present significantly decreased the vascular density compared to WT mice (27). Fib-3 deficiency did not alter the overall presence of cardiomyocytes within CSs.

In our study, we demonstrated major differences between Fib-3 KO and WT CSs following I/R injury *in vitro*. We found that both WT and Fib-3 KO CSs respond similarly to intracellular changes in O_2_ concentrations. Our findings showing that I/R-induced reduction in cell viability was rescued in Fib-3 KO CSs are consistent with the reduction in oxidative stress and correlated protection against vascular smooth muscle cell calcification observed in other Fib-3 KO cells (7). We found no difference in contractional frequency of non-infarcted WT and Fib-3 KO CS whereas fractional shortening significantly decreases at baseline in Fib-3 KO CS compared to WT CS. Consistent with our previous study of I/R CSs, fractional shortening and contraction frequency was reduced in both WT and Fib-3 KO I/R CSs when compared to their respective controls (1). However, it seems like Fib-3 deficiency in CSs might be protective against I/R injury-induced reduction in contraction frequency, while no significant difference was measured in fractional shortening.

Our qPCR analyses demonstrated that overall changes in relative expression of genes involved in cardiovascular disease measured in Fib-3 KO I/R CSs were presenting a similar trend with changes measured in WT I/R CSs when compared to their relative controls, similar to what we previously reported in CSs (1). However, these were significantly different when Fib-3 KO I/R CSs were compared to WT I/R CSs. In this study, sarcoplasmic genes were significantly different among non-infarcted WT and Fib-3 KO CSs. However, Fib-3 KO I/R CSs showed non-significant differences in fold change values of all sarcoplasmic genes compared to WT I/R CSs, suggesting that Fib-3 deficiency does not play a role on sarcomeric proteins following I/R. We found that Actc1 was downregulated in both WT and Fib-3 KO I/R CSs compared to their respective controls. Actc1 mutation was previously reported to upregulate Fib-3 upregulation, supporting a potential correlation between Fib-3 deficiency and sarcomeric proteins regulating contractile function (28). With respect to calcium ion transport genes, we found a significant increase in relative expression of Atp5a1 in Fib-3 KO I/R CSs compared to WT I/R CSs, which may play a role in regulating contractile function and response to I/R injury. In terms of other genes evaluated in this study, a previous study demonstrated a role played by Fib-3 on Ccnd1 expression levels in lung cancer cells (29). In our study we observed that Ccnd1 expression was dependent on Fib-3 when comparing KO I/R CSs to WT I/R CSs. Ace was overexpressed in Fib-4 deficient mice (30). Fib-3 deficiency increases Ace gene expression levels following I/R injury in CSs. Owing to structural similarities between Fib-3 and Fib-4 and supported by our observations, these data suggest a role played by Fib-3 on Ace expression levels.

Mmp inhibitor including Fib-3, play a positive role in mediating angiogenesis (31). Most commonly angiogenic assays are based on the use of HUVECs (tube formation assay, spheroid-based sprouting assay or aortic ring assays) (32–34). In our study we have focused on the endothelial cell network formed within 3D CSs as *in vitro* complex models of the human heart, which enabled us to study the role of Fib-3 deficiency on Mmps in 3D CSs. Previous reports did not clearly identify if Fib-3 plays a positive or negative role on angiogenesis. For instance, Zhongwei et al. (23) reported that Fib-3 may inhibits Mmp2/9 and improves vascular health of spontaneously hypertensive rats. In contrast, Wang et al. (35) demonstrated that Fib-3 promotes angiogenesis and contributes to development of psoriasis by increasing the expression of vascular endothelial growth factor (VEGF) in ECs. Nandhu et al. (36) reported that silencing Fib-3 in glioblastoma cells downregulates the expression of Mmp9 and Mmp13. In our study, we demonstrated a significant decrease in Mmp13 in non-infarcted Fib-3 KO CSs compared to WT CSs whereas a significant upregulation of Mmp13 was observed in Fib-3 KO I/R CSs compared to WT I/R CSs, supporting a positive effect of Fib-3 deficiency on angiogenesis following I/R. Ren1, another cardiac remodeling gene, was also upregulated in both WT and Fib-3 KO I/R CSs compared to control KO I/R CSs. However, Ren1 expression was significantly decreased in Fib-3 KO I/R CSs compared to WT I/R CSs. Altogether our findings suggest that reduction in cardiac remodeling genes expression in Fib-3 KO I/R CSs may also promote angiogenesis.

Our qPCR analysis of apoptosis regulating genes such as Ccl11, Mao, Nppb and Npr1 showed a significantly decreased in Fib-3 KO I/R CSs compared to WT I/R CSs, suggesting a protective role of Fib-3 deficiency against I/R mediated apoptosis. This is consistent with a previous study where Fib-3 downregulation in cutaneous squamous cell showed protective effect against drug-induced apoptosis (37).

Fib-3 degrades collagen type 2 (Col2) whereas suppression of Fib-3 significantly increased Col2 in mice and rats (38, 39). In contrast in our study, we have showed that Ctgf, Col11a1, Col1a1 were significantly downregulated and Col3a1 and F2r were significantly upregulated in Fib-3 KO CS compared to WT CSs. Whereas, Ctgf, Col11a1, Col1a1 were upregulated in Fib-3 KO I/R CSs compared to WT I/R CSs, supporting a role played by Fib-3 on fibrotic scar formation. Fib-3 presents a narrow binding spectrum and Fn1 does not bind to Fib-3 in mice (13). However, we report that both F2r and Fn1 didn't present any difference in their expression in non-infarcted and I/R Fib-3 KO CSs.

In our study we evaluated the direct relationship between adrenergic receptor proteins functions and Fib-3 following IR injury. We demonstrated that Adra1a, Adrb2 and Adrb3 were significantly downregulated and Adra1b was significantly upregulated in Fib-3 KO I/R CSs compared to WT I/R CSs. Adrenergic receptor proteins alpha 2a (Adra2a) inhibits the activation of PI3K/Akt/mTOR pathway in cervical cancer cells (40) which suggests that there could be a direct relation between adrenergic receptor protein functions and Fib-3. Previous studies report a role played by Fib-3 on human cervical cancer cell growth and metastasis *in vitro* and *in vivo* via PI3K-Akt-mTOR signal transduction pathway (41). We also observed that Map2k5 and Mapk8 expression were significantly downregulated in Fib-3 KO I/R CSs compared to WT I/R CSs, consistent with previous reports about the role played by Fib-3 on activate mitogen-activated protein kinases (42).

To our knowledge, our study using our previously established model of 3D *in vitro* CSs is the only one that investigated the role of Fib-3 deficiency in myocardial I/R injury from the morphological, cellular, and extracellular level. In our study, we demonstrated that Fib-3 deficiency alters the expression of genes regulating cardiac remodeling, angiogenesis, and cardiac fibrosis following I/R injury. Since Fib-3 is an ECM component, in future we will study the effect of ECs on other cardiac cells by combining Fib-3 KO CSs with ECs. We will also evaluate CSs for the parameters like structure of matrix, ECM deposition and stiffness, and staining for sirius red and caspase-3. Given its emerging role in cardiac pathophysiology, future studies may also aim at comparing the effects of Fib-3 using human cells in 3D CSs and hopefully be supported by *in vivo* animal studies comparing Fib-3 KO and WT animals. This may further unveil the role played by Fib-3 as a potential therapeutic target for MI patients.

## Conclusion

In this study we showed that Fib-3 deficiency is protective against I/R injury in 3D *in vitro* neonatal mouse CSs. Future studies may aim at better understanding the role played by Fib-3 in mediating cardiac fibrosis and angiogenesis in animal models and potentially translate findings from the bench to the bedside for patients suffering from MI-induced cardiac fibrosis.

## Data Availability Statement

The original contributions presented in the study are included in the article/Supplementary Materials, further inquiries can be directed to the corresponding author.

## Ethics Statement

The animal study was reviewed and approved by Animal Ethics Committee at the Northern Sydney Local Health District St Leonards, NSW, Australia.

## Author Contributions

PS contributed to the conceptualization, data generation, data curation, data analysis, data visualization, funding acquisition, investigation, methodology, resources, validation, project administration, writing original draft, manuscript review, and editing. DB contributed to data analysis and manuscript review. LM contributed for manuscript review and editing. AB and GF were responsible for the supervision, manuscript review, and editing. CG contributed to the conceptualization, data generation, data curation, data analysis, data visualization, funding acquisition, methodology, project administration, supervision, manuscript review, and editing. All authors contributed to the article and approved the submitted version.

## Funding

PS was supported by University of Newcastle with UNIPRS and UNRS Central and Faculty School (UNRSC5050) scholarships. CG was supported by a UTS Seed Funding, Catholic Archdiocese of Sydney Grant for Adult Stem Cell Research and a University of Sydney/Sydney Medical School Foundation Cardiothoracic Surgery Research Grant.

## Conflict of Interest

The authors declare that the research was conducted in the absence of any commercial or financial relationships that could be construed as a potential conflict of interest.

## Publisher's Note

All claims expressed in this article are solely those of the authors and do not necessarily represent those of their affiliated organizations, or those of the publisher, the editors and the reviewers. Any product that may be evaluated in this article, or claim that may be made by its manufacturer, is not guaranteed or endorsed by the publisher.
