# Supplementary material for: Fibulin-3 Deficiency Protects Against Myocardial Injury Following Ischaemia/ Reperfusion in in vitro Cardiac Spheroids
Source: Front Cardiovasc Med. 2022 Jun 20;9:913156. doi: 10.3389/fcvm.2022.913156 (PMC9251181; doi:10.3389/fcvm.2022.913156)
Supplement: Supplementary file 7 [file Data_Sheet_1.pdf]

SUPPLEMENTARY MATERIALS

Supplemental material legends

Movie 1 is a 3D rendering analysis of collapsed Z-stack confocal image of a WT CS stained with antibodies against CD31 for endothelial cells (green), cTNT for cardiomyocytes (red) and Hoechst for nuclei (blue). It showed the 3D orientation of endothelial cell network within the CS.

Movie 2 is a 3D rendering analysis of collapsed Z-stack confocal image of a Fib-3 KO CS stained with antibodies against CD31 for endothelial cells (green), cTNT for cardiomyocytes (red) and Hoechst for nuclei (blue). In contrast to 3D rendering analysis of WT CS (Movie 1), Fib-3 KO CS showed a reduced CD31-positive endothelial cells in 3D orientation.

Movie 3 is showing the contractile activity of a control WT CS recorded over the IonOptix system.

Movie 4 is showing the cessation of contractile activity in a WT CS following I/R injury recorded over the IonOptix system.

Movie 5 is showing the comparison between the contractile activity of a control WT CS (Movie 3) and Fib-3 KO CS recorded over the IonOptix system. It showed that Fib-3 deficiency in a CS increases its contractional frequency.

Movie 6 is showing contractile activity of a Fib-3 KO I/R CS recorded over the IonOptix system. It demonstrated that compared to WT I/R CS (Movie 4), Fib-3 KO I/R CS were able to retain their contractile activity.

Figure 1. Changes in Cytoplasmic Genes in Non-infarcted WT and Fib-3 KO CSs. Relative expression of Actc1, Myh10, Myh6, TNNi3 and Tnnt2 in WT and Fib-3 KO CSs. Unpaired t-test, $p>0.05$ = ns, $p<0.05$ = *, $p<0.001$ = *** and $p<0.0001$ = ****. Error bars represent the mean ± SD (n = 4).

Figure 2. Changes in Cytoplasmic Genes in WT and Fib-3 KO CSs Following I/R Injury. Relative expression of Actc1, Myh10, Myh6, TNNi3 and Tnnt2 in WT and Fib-3 KO CSs. Two-way ANOVA with Sidak's multiple comparison test, $p>0.05$ = ns. Error bars represent the mean ± SD (n = 4).

Figure 3. Changes in Calcium Ion Transport Genes in WT and Fib-3 KO CSs Following I/R Injury. Relative expression of Atp2a2 and Atp5a1 in WT and Fib-3 KO CSs. Two-way ANOVA with Sidak's multiple comparison test, $p<0.05$ = *, $p<0.01$ = **, $p<0.001$ = *** and $p<0.0001$ = ****. Error bars represent the mean ± SD (n = 4).

Figure 4. Changes in Cell Cycle-regulating Genes in WT and Fib-3 KO CSs Following I/R Injury. Relative expression of Ccnd1 and Rarres1 in WT and Fib-3 KO CSs. Two-way ANOVA with Sidak's multiple comparison test, $p>0.05$ = ns and $p<0.01$ = **. Error bars represent the mean ± SD (n = 4).

Figure 5. Changes in Adrenergic Receptor Proteins in WT and Fib-3 KO CSs Following I/R Injury. Relative expression of Adra1a, Adra1b, Adra1d, Adrb2 and Adrb3 WT and Fib-3 KO CSs. Two-way ANOVA with Sidak's multiple comparison test, p>0.05 = ns, p<0.01 = ** and p<0.0001= ****. Error bars represent the mean ± SD (n = 4).

Figure 6. Changes in Map Kinases in WT and Fib-3 KO CSs Following I/R Injury. Relative expression of Map2k5, Mapk1 and Mapk8 WT and Fib-3 KO CSs. Two-way ANOVA with Sidak's multiple comparison test, p>0.05 = ns, p<0.01 = ** and p<0.0001= ****. Error bars represent the mean ± SD (n = 4).

Figure 7. Changes in Phosphodiesterases in WT and Fib-3 KO CSs Following I/R Injury. Relative expression of Pde3a, Pde3b, Pde5a and Pde7a in WT and Fib-3 KO CSs. Two-way ANOVA with Sidak's multiple comparison test, p>0.05 = ns, p<0.05 = * and p<0.0001= ****. Error bars represent the mean ± SD (n = 4).

Figure 8. Difference in Spheroid Formation Rate of WT and Fib-3 KO. (A) Representative images of WT and Fib-3 KO CSs at day 4. (B) Statistical analyses of CS formation in WT versus Fib-3 KO CSs. Unpaired t-test, p<0.0001 = ****. Error bars represent the mean ± SD (n =384).
