# Supplementary figures and images for: Fibulin-3 Deficiency Protects Against Myocardial Injury Following Ischaemia/ Reperfusion in in vitro Cardiac Spheroids
Source: Front Cardiovasc Med. 2022 Jun 20;9:913156. doi: 10.3389/fcvm.2022.913156 (PMC9251181; doi:10.3389/fcvm.2022.913156)

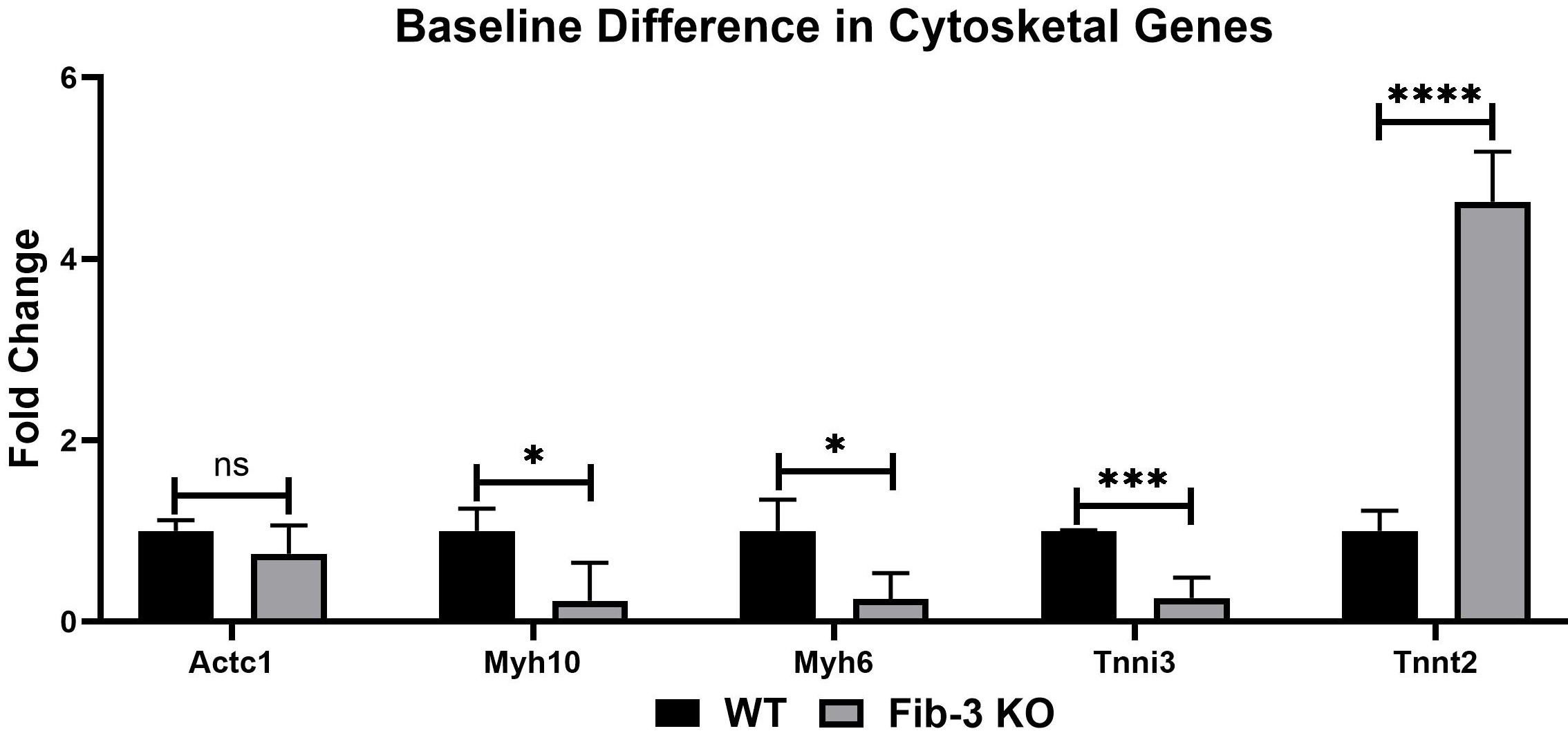

Supplement: Supplementary file 8 [file Image_1.JPEG]

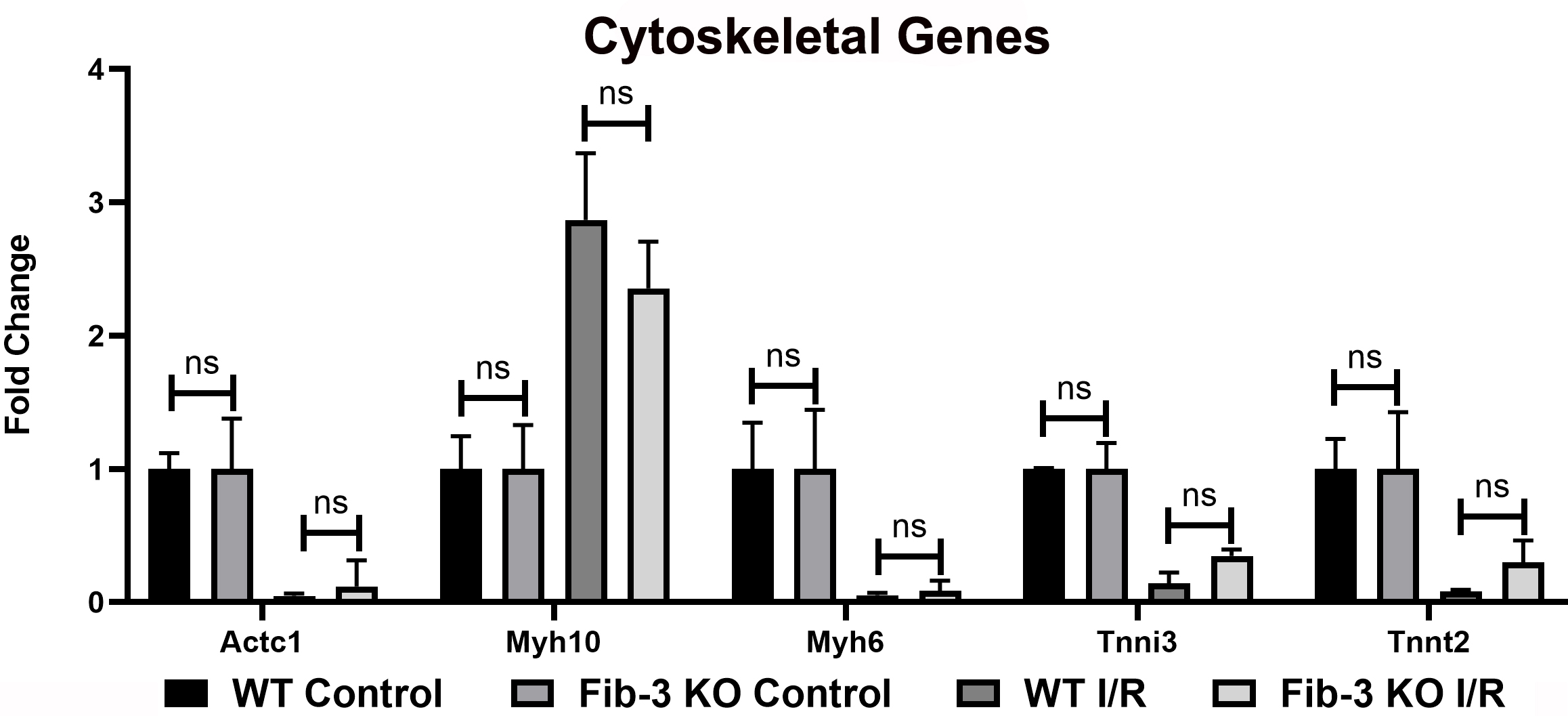

Supplement: Supplementary file 9 [file Image_2.JPEG]

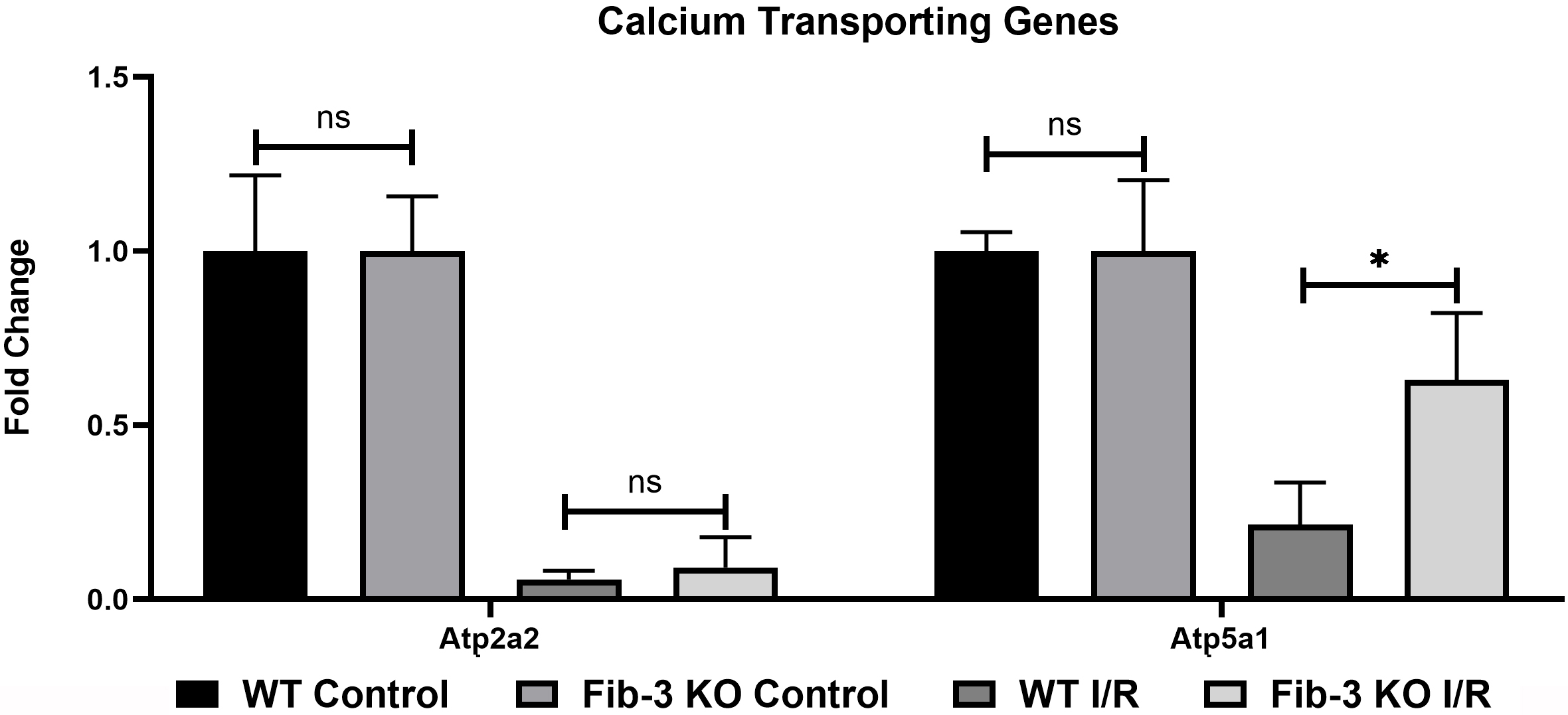

Supplement: Supplementary file 10 [file Image_3.JPEG]

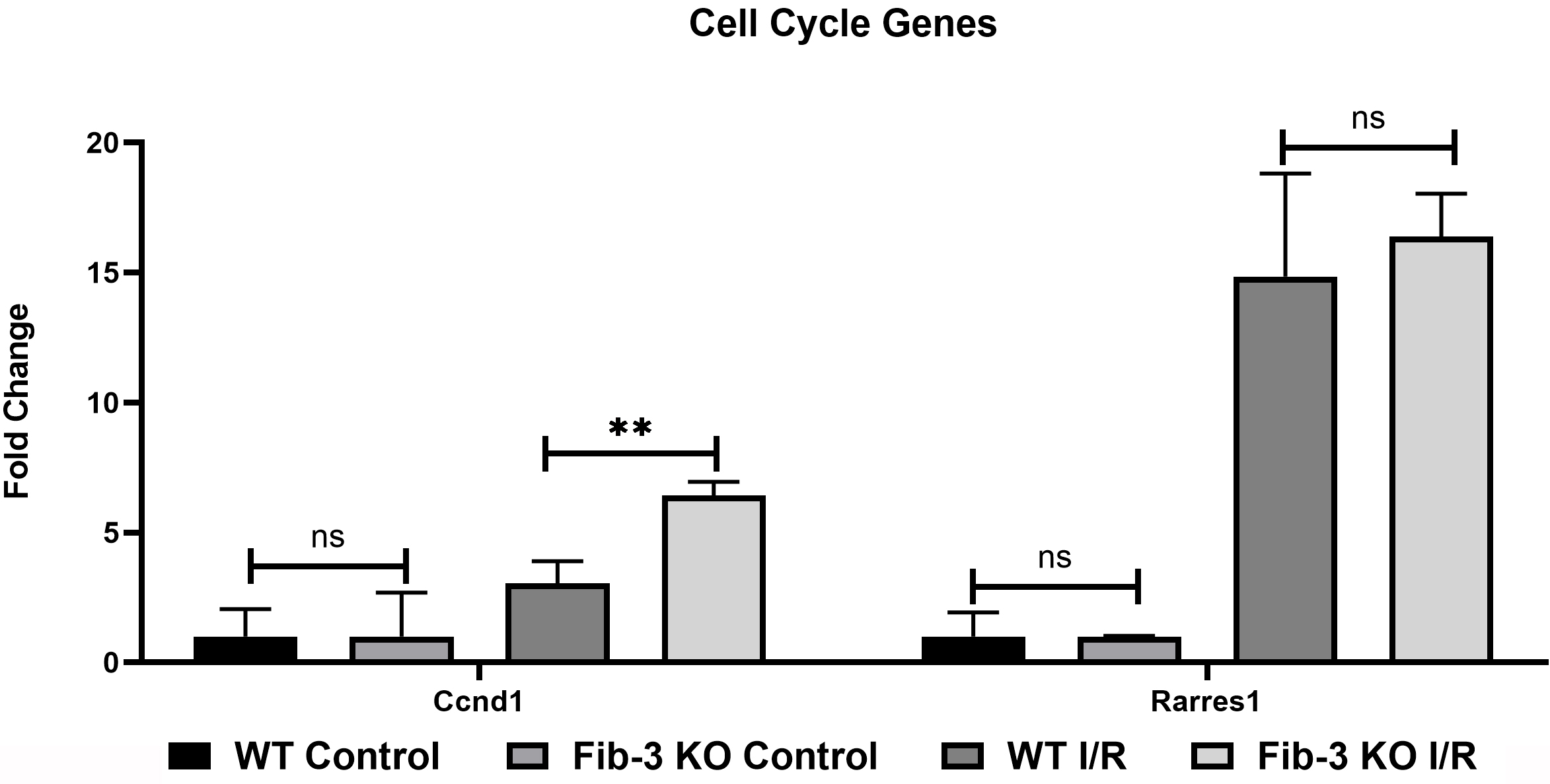

Supplement: Supplementary file 11 [file Image_4.JPEG]

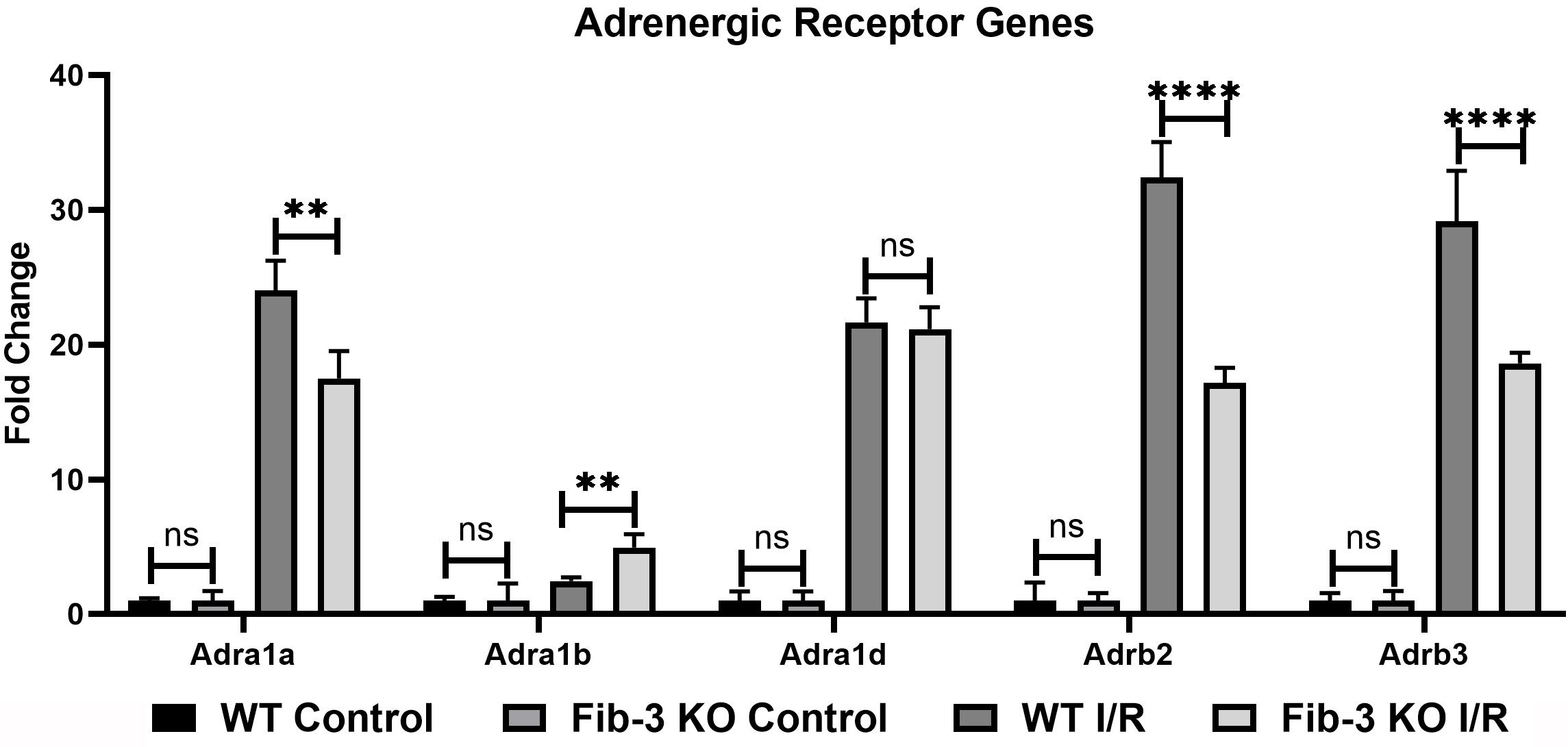

Supplement: Supplementary file 12 [file Image_5.JPEG]

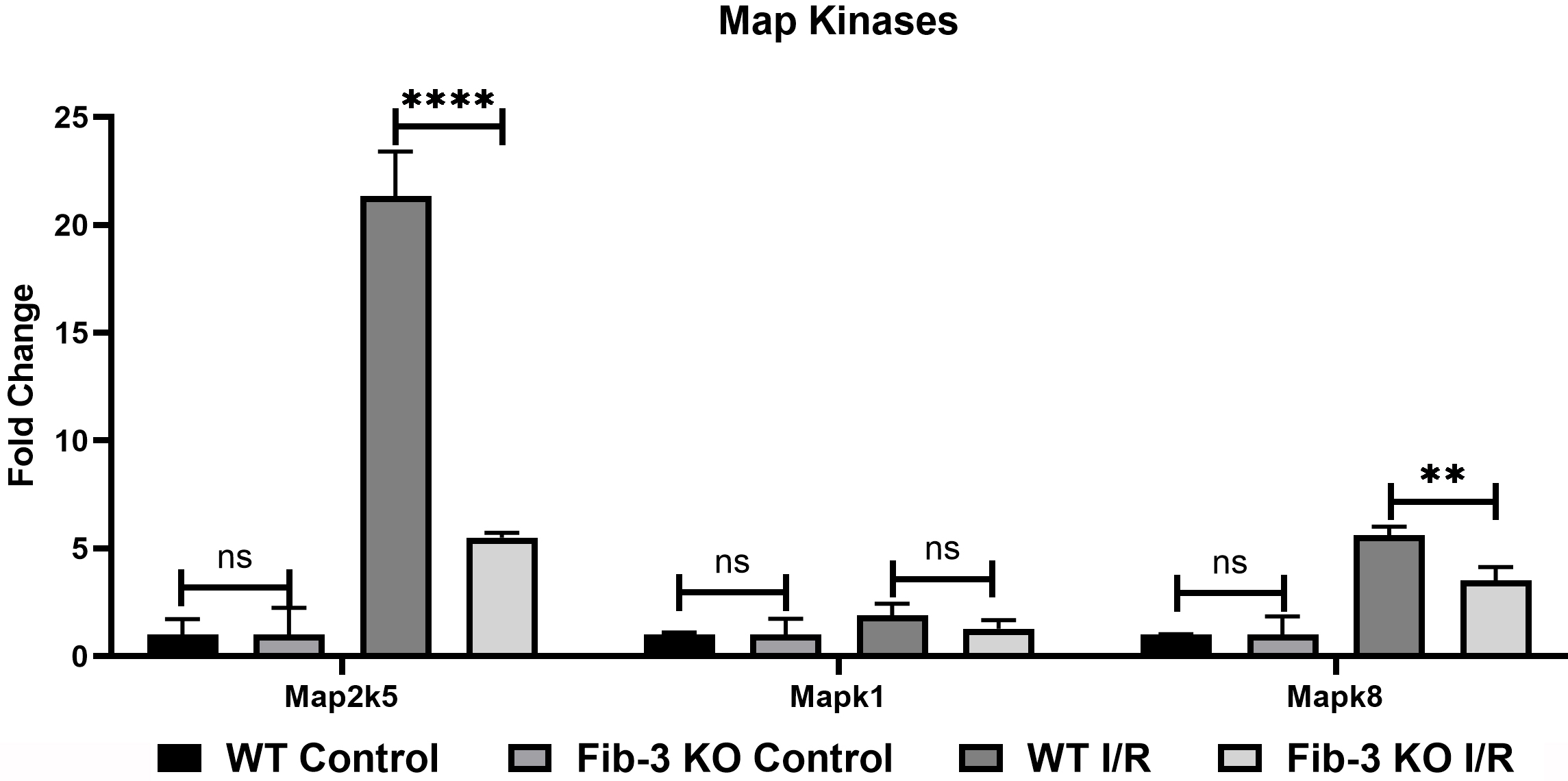

Supplement: Supplementary file 13 [file Image_6.JPEG]

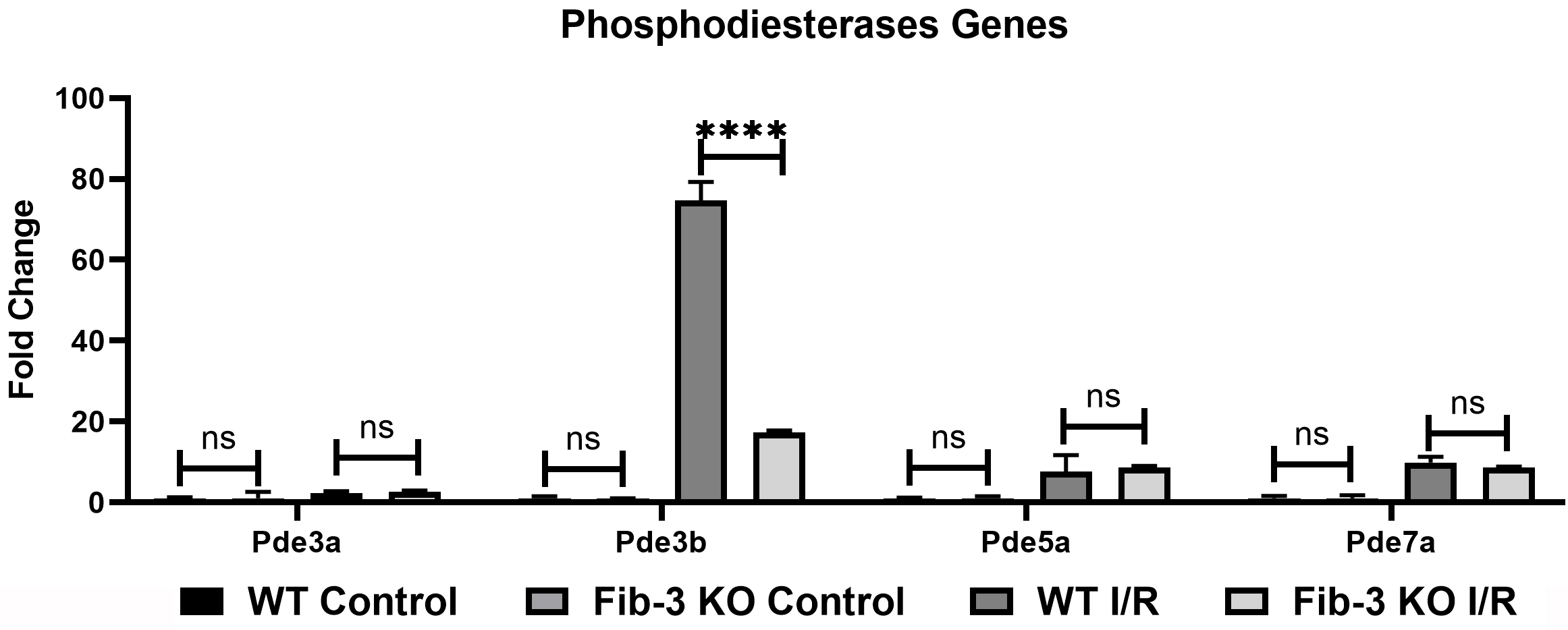

Supplement: Supplementary file 14 [file Image_7.JPEG]

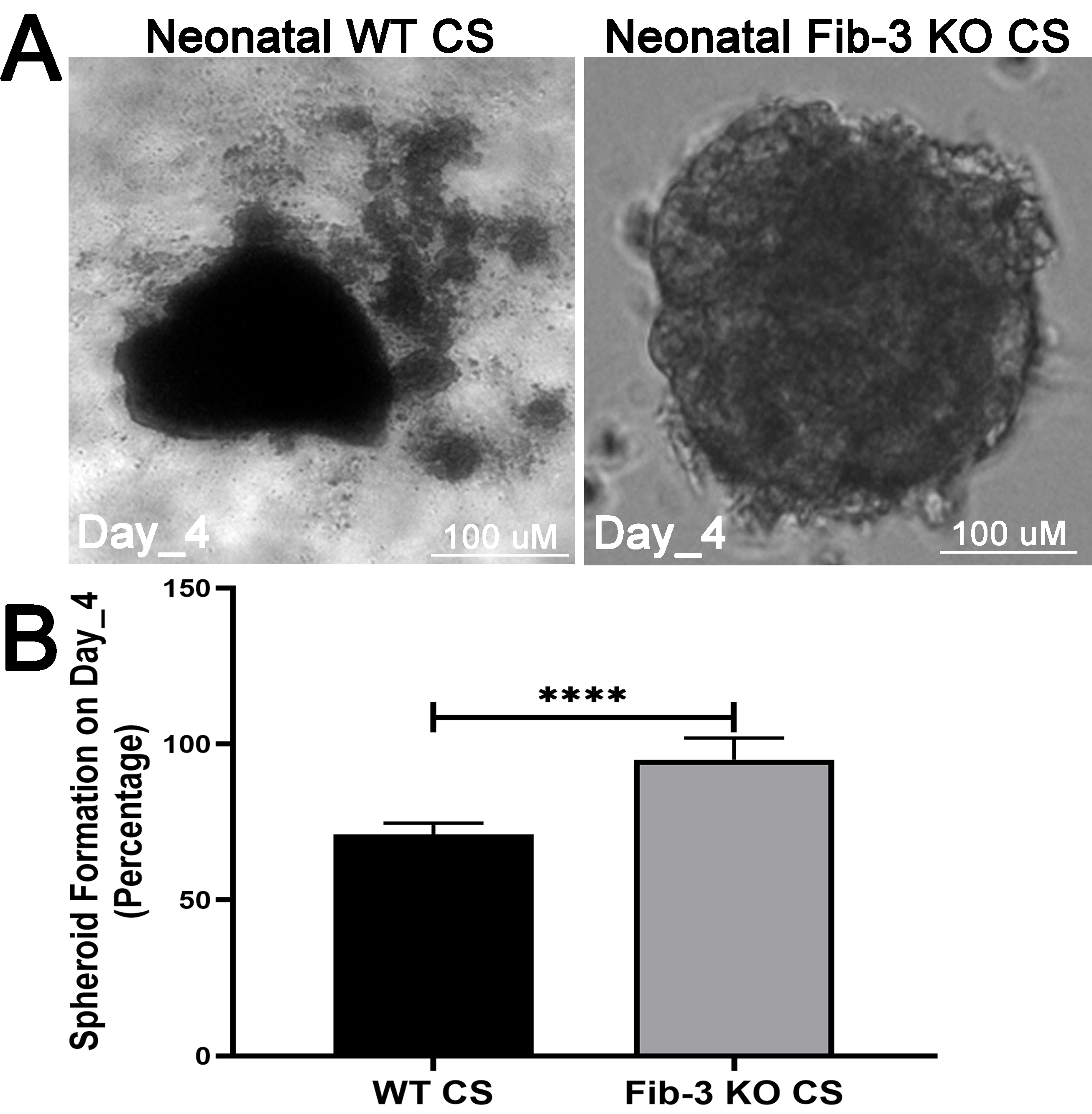

Supplement: Supplementary file 15 [file Image_8.JPEG]
